# A Novel Small Molecule Inhibits Intrahepatocellular Accumulation of Z-Variant Alpha 1-Antitrypsin In Vitro and In Vivo

**DOI:** 10.3390/cells8121586

**Published:** 2019-12-06

**Authors:** Xiaojuan Zhang, Kien Pham, Danmeng Li, Ryan J. Schutte, David Hernandez Gonzalo, Penghui Zhang, Regina Oshins, Weihong Tan, Mark Brantly, Chen Liu, David A. Ostrov

**Affiliations:** 1 Department of Pathology, Immunology and Laboratory Medicine, University of Florida College of Medicine, Gainesville, FL 32611, USA; sophiezhang@ufl.edu (X.Z.); danmeng1986@ufl.edu (D.L.); rschutte@pathology.ufl.edu (R.J.S.); hernand3@ufl.edu (D.H.G.); 2 Department of Pathology and Laboratory Medicine, New Jersey Medical School, Rutgers, The State University of New Jersey, Newark, NJ 07103, USA; ktp52@njms.rutgers.edu; 3 Center for Research at Bio/Nano Interface, Department of Chemistry and Department of Physiology and Functional Genomics, UF Health Cancer Center, UF Genetics Institute and McKnight Brain Institute, University of Florida, Gainesville, FL 32611, USA; phzhang@xjtu.edu.cn (P.Z.); tan@hnu.edu.cn (W.T.); 4 The Key Laboratory of Biomedical Information Engineering of the Ministry of Education, School of Life Science and Technology, Bioinspired Engineering and Biomechanics Center (BEBC), Xi’an Jiaotong University, Xi’an 710049, China; 5 Division of Pulmonary, Critical Care, and Sleep Medicine, University of Florida College of Medicine, Gainesville, FL 32610, USA; Regina.Oshins@medicine.ufl.edu (R.O.); Mark.Brantly@medicine.ufl.edu (M.B.)

**Keywords:** 4′,′5-(Methylenedioxy)-2-Nitrocinnamic Acid, alpha-1 antitrypsin, SERPINA1*E342K, PiZ mouse, molecular docking

## Abstract

Alpha 1-antitrypsin deficiency (AATD) is the most common genetic cause of liver disease in children and is associated with early-onset chronic liver disease in adults. AATD associated liver injury is caused by hepatotoxic retention of polymerized mutant alpha 1-antitrypsin molecules within the endoplasmic reticulum. Currently, there is no curative therapy for AATD. In this study, we selected small molecules with the potential to bind mutant alpha 1-antitrypsin (Z-variant) to inhibit its accumulation in hepatocytes. We used molecular docking to select candidate compounds that were validated in cell and animal models of disease. A crystal structure of polymerized alpha 1-antitrypsin molecule was used as the basis for docking 139,735 compounds. Effects of the top scoring compounds were investigated in a cell model that stably expresses Z-variant alpha 1-antitrypsin and in PiZ mice expressing Z-variant human alpha 1-antitrypsin (Z-hAAT), encoded by SERPINA1*E342K. 4′,′5-(Methylenedioxy)-2-nitrocinnamic acid was predicted to bind cleaved alpha 1-antitrypsin at the polymerization interface, and observed to co-localize with Z-hAAT, increase Z-hAAT degradation, inhibit intracellular accumulation of Z-hAAT, and alleviate liver fibrosis.

## 1. Introduction

Alpha 1-antitrypsin (AAT) is a serum glycoprotein primarily synthesized in hepatocytes that functions as a protease inhibitor with targets including neutrophil proteases released during inflammation [1]. Structurally, alpha 1-antitrypsin is comprised of three β-sheets and nine α-helices, with the reactive center loop at the apex of the domain [2]. The reactive center loop and β-sheet A are flexible, which is required for protease inhibition [3]. In AAT deficiency (AATD), the mutant AAT molecules, a β-strand formed by cleavage of the reactive center loop readily inserts into β-sheet A of a second molecule to form a dimer, which then extends into polymeric chains and aggregates [4]. Accumulation of the Z-variant AAT (the most frequent and severe mutation, encoded by SERPINA1*E342K) in the endoplasmic reticulum of liver cells has a gain of function proteotoxic effect on the liver, resulting in fibrosis, cirrhosis and/or hepatocellular carcinoma [5]. Low concentrations of serum AAT are detrimental since there is insufficient protection to the lung tissues from attack by neutrophil proteases [5]. Currently, liver transplantation is the only effective treatment for end-stage liver disease [6] and expensive AAT augmentation therapy is used for lung disease [6]. Since it is essential to develop new strategies to treat AATD and associated diseases, therapeutic strategies are under development to reduce AAT accumulation in the liver and enhance AAT secretion into the blood. 

Expression of the Z-variant human AAT gene (Z-hAAT) in transgenic mice (PiZ) demonstrates that accumulation of proteotoxic Z-hAAT at hepatic sites is the primary mechanism for liver disease [7]. PiZ mice develop intrahepatic Periodic Acid–Schiff diastase (PASD) resistant globular inclusions that are the histological hallmark of AATD [7]. In addition, PiZ mice develop hepatocyte pathology that resembles diseased liver in humans with AATD [7,8]. In our previous study, a novel PiZ hepatocyte culture system was developed to study the kinetics of Z-hAAT production, polymerization, retention, and cellular response [9]. This novel system also serves as a platform to identify and characterize novel therapeutics capable of mitigating proteotoxicity [9].

Previous studies proposed developing drugs to modulate conformational transitions of AAT [10,11,12,13,14,15]. However, limited efficacy has been shown in animal models and no promising activity has been reported in humans. In this study, we attempted a novel strategy, to target the interface of AAT involved in polymerization with drug-like small molecules. The crystal structure of AAT polymer (PDB code 1QMB) [4] was the basis for selection of drug-like compounds from 139,735 small molecules in the National Cancer Institute Developmental Therapeutics repository (2007 plated set). Top scoring compounds were tested for activity against aggregation of Z-hAAT in an in vitro PiZ hepatocyte culture system and in vivo in PiZ mice.

## 2. Materials and Methods

### 2.1. Molecular Docking to Identify Candidate Compounds

The crystal structure of AAT (the cleaved AAT polymer, 1QMB [4]) provided the basis for molecular docking. The interface of AAT involved in polymerization, consisting of residues located in strand 4A, strand 3A, and helix F (positions 177–185 and 328–352 in 1QMB), was selected as the molecular docking target. The DOCK6 (UCSF) program package was used to prepare files and to run molecular docking by parallel processing at the University of Florida High Performance Computing Center. SPHGEN was used to identify the locations of potential ligand atoms. GRID was used to generate scoring grids estimating polar and non-polar interactions (for van der Waals and electrostatic scores). The screened compound library contained the atomic coordinates for 139,735 compounds in the NCI DTP 2007 plated set, obtained from ZINC (UCSF). The top 20 scoring compounds (based on the overall Energy Score, summing polar and non-polar interactions) were obtained from a repository at the NIH National Cancer Center Developmental Therapeutics Program [16] for testing in vitro. 4′,′5-(Methylenedioxy)-2-nitrocinnamic acid (CAS: 6315-90-8, purity: 98%) was purchased commercially (Alfa Asesar, Haverhill, MA, USA) to evaluate in vitro effects and in vivo studies.

### 2.2. Cell Culture and Compound Treatment

Z-hAAT mouse malignant hepatocytes [9] were cultured in advanced DMEM/F12, 10% fetal bovine serum, 6 mM l-glutamine, 1% penicillin/streptomycin, and 40 ng/mL dexamethasone in 5% CO_2_ at 37 °C. Cultures of the human HepG2 hepatoma cell line (ATCC, Manassas, VA, USA) were similarly maintained.

Z-hAAT hepatocytes (1–2 × 10^4^) and HepG2 cells (6–8 × 10^4^) were seeded on 96-well tissue culture plates. Twenty-four hours post-plating, stock preparations of compounds dissolved in DMSO (Dimethyl sulfoxide) were diluted in culture medium to test dosage (0.1% DMSO) and were added to the wells. After 24–48 h treatment, the cell lysate and supernatant were collected for ELISA. 

For cultures assayed to determine protein degradation, proteosome inhibitor (MG132, 30 μM) (Selleck Chemical LLC, Houston, TX, USA), lysosomal inhibitors (E64D (Bachem Americas, Inc., Torrance, CA, USA), and pepstatin A (Affymetrix, Inc., Santa Clara, CA, USA) at 20 μg/mL) were added 6 h and 4 h before harvesting from six-well plates [17]. All in vitro experiments were conducted three times.

### 2.3. Cell Proliferation and Cytotoxicity Assay

Z-hAAT hepatocytes (5 × 10^3^) and HepG2 cells (4 × 10^4^) were seeded on 96-well tissue culture plates. Twenty-four hours post-plating, 100 µL of diluted compounds were added to triplicate wells. CellTiter Aqueous One Solution Cell Proliferation Assay kit (Promega, G3580, Madison, WI, USA) was used to determine cell proliferation and cytotoxicity 72 h after treatment.

### 2.4. Heat Induced Polymerization Assay

Purified Z-AAT (2 µg) obtained from PiZZ patients’ serum was incubated alone, with DMSO, or with UFC1 in 4.75% (v/v) DMSO (50 and 100 molar fold of Z-AAT) at 41 °C for seven days. The protein was then run on a 7.5% (w/v) nondenaturing PAGE gel for 2 h at 100 V on ice. Wild-type AAT (Purified from PiMM health control serum) and compound CG [18] were used as no polymerization and inhibition on heat-induced polymerization control, respectively. 

### 2.5. Enzyme-Linked Immunosorbent Assay

Immuno Plates (Fisher Scientific International, Inc, Pittsburgh, PA, USA) were coated with 50 µL goat anti-human alpha-1 antitrypsin antibody (Table S1) at 4 °C overnight. The plate was washed three times with 1× PBST (phosphate buffered saline with 0.05% Tween-20) and then blocked with 100 µL 3% bovine serum albumin (BSA) (Sigma-Aldrich, St. Louis, MO, USA) for 1 h. After blocking, 50 µL samples were loaded in triplicate wells and incubated for 1 h at 37 °C. After washing with 1× PBST, the rabbit anti-human alpha 1-antitrypsin antibody (Table S1) was added and incubated for 1 h at 37 °C and then washed three times with 1× PBST. Goat-anti rabbit HRP antibody (Table S1) was added to the wells and incubated at 37 °C for 1 h. After a final wash with 1× PBST, o-phenylenediamine tablets (Sigma-Aldrich, St. Louis, MO, USA) were dissolved in ddH_2_O and 50 μL solutions were added to the wells to develop color. H_2_SO_4_ (10%, Sigma-Aldrich, St. Louis, MO, USA) was added to terminate color development. The absorbance was measured at a wavelength of 490 nm in an ELISA plate reader (Molecular Devices, LLC, CA, USA). 

### 2.6. Luciferase Assay 

Human AAT (hAAT) knockout Huh 7.5 hepatoma cells cultured in DMEM/F12 supplemented with 10% fetal bovine serum and antibiotic were transfected with a tetracycline (Tet) inducible luciferase-tagged Z-hAAT plasmid using X-tremeGENE HP DNA transfection reagent in a 96-well plate. After 12 h, cells were treated in triplicate with compounds suspended in DMSO at concentrations ranging from 1 nM to 1 μM using media supplemented with tetracycline to turn on Z-hAAT plasmid expression. Media was collected at 24 h post-treatment and luciferase activity was measured to determine the amount of secreted Z-hAAT. Cell viability was determined prior to the assay. 

### 2.7. Animal Experiments

PiZ transgenic mice were kindly provided by Dr. Jeffery Teckman’s laboratory at St. Louis University and were bred in the animal facility of University of Florida. The mice were housed under controlled temperature and humidity with a 12:12 h light–dark cycle. Water and food were available ad libitum. Eight-week old female PiZ mice were randomly divided into two-week (n = 6 both in treated and control groups), one-month (n = 5 in control group, n = 6 in treated group) and three-month (n = 7 both in treated and control groups) compound treated and control groups. Compounds were dissolved in DMSO (final concentration 1% v/v), diluted in PBS, and delivered by oral gavage daily. The control mice were given equal amounts of DMSO with PBS as the treated cohort. Body weight was measured and animal behavior was observed daily. Blood samples were collected at baseline and monthly for three months. Serum was separated by centrifuge at 250 g for 10 min. The mice were euthanized with CO_2_ at the endpoint. Liver tissues were harvested for IHC, immunofluorescence, picrosirius red stain, and molecular analysis. Major organs were collected for Hematoxylin and eosin staining including liver, heart, lung, kidney, spleen, and pancreas. The protocol #201509202 was approved on 5 February 2018 by the Institution of Animal Care and Use Committee of University of Florida.

### 2.8. Periodic Acid–Schiff Diastase (PASD) Stain

The liver tissues were fixed in 10% formalin and embedded in paraffin. Tissue sections (4 μm) were de-paraffinized and rehydrated with water. The slides were put in an amylase solution for 20 min at room temperature before placing in 0.5% periodic acid (Sigma-Aldrich) for 10 min, and then Schiff’s reagent (Thermo Fisher) was added for 15 min after washing.

### 2.9. Immunohistochemistry (IHC) Stain

Slides were prepared as above and treated by Citra Steam (Biogenex, Fremont, CA, USA) for 30 min and 2.5% normal goat serum (Vector labs) for 20 min. The slides were incubated with primary antibody (Table S1) for 60 min. Secondary antibody (Table S1) was applied after washing. Stain was visualized using the DAB (3,3′-Diaminobenzidine) chromagen (Vector Laboratories, Burlingame, CA, USA) and CAT hematoxylin counterstain (Biocare Medical, Walnut Creek, CA, USA). Slides were scanned (Aperio Scanscope CS, Leica Biosystems, Wetzlar, Germany) and data were analyzed by ImageScope software (Leica Biosystems, Wetzlar, Germany).

### 2.10. Picrosirius Red Stain

Picrosirius red stain was performed with a commercially available kit (Polysciences, Inc., Warrington, PA, USA). Slides were deparaffinized and hydrated in distilled water. Slides were then incubated in solution A for 2 min, rinsed with distilled water, incubated in solution B for 60 min, transferred to solution C for 2 min, and then transferred to 70% ethanol for 45 s. Slides were then dehydrated, cleared, and mounted. Slides were scanned (Aperio Scanscope CS, Leica Biosystems, Wetzlar, Germany) and data analyzed by ImageScope software (Leica Biosystems, Wetzlar, Germany). The stain of blood vessels was excluded from analysis.

### 2.11. Double Stain Immunofluorescence

Double stain immunofluorescence was used to detect biotin conjugated compounds interaction with hAAT protein in the cells and the expression of hAAT polymers and autophagosomes (LC3 positive stain) in PiZ mice livers.

In brief, cells were seeded on Poly-d-lysine coated coverslip in six-well plates. Twenty-four hours after treatment with biotin conjugated (Appendix A) or non-conjugated UFC1 at 10 μM, cells were washed and blocked with 10% normal goat serum (Vector labs) for 20 min. Rabbit anti-human AAT primary antibody (Table S1) was applied and incubated at 4 °C overnight. After washing, goat anti-rabbit (Alexa Fluor 488) and streptavidin conjugated with tetramethylrhodamine secondary antibodies were added to the cells. For biotin positive control, goat anti-IgG antibody conjugated with biotin was used instead of goat anti-rabbit Alexa Fluor 488. Cell viability and biotin conjugated compounds function were determined prior to the double stain study.

Paraffin slides were prepared as above. After antigen retrieval, slides were incubated with anti-mouse human AAT polymer (Table S1) and anti-mouse LC3 antibodies (Table S1) at 4 °C overnight. After washing, secondary antibodies (Table S1) were applied at room temperature for 60 min before DAPI (4′,6-diamidino-2-phenylindole) nuclear staining (Thermo fisher, Waltham, MA, USA).

Slides were covered with anti-fade mounting medium before visualization on an immunofluorescence microscope (Zeiss, Axioskop 2 mot plus, Oberkochen, Germany). Data were analyzed by Image J (National Institutes of Health).

### 2.12. Alanine Transaminase and Aspartate Aminotransferase Activity Assay

Alanine transaminase (ALT) and Aspartate aminotransferase (AST) assay kits were purchased from Sigma-Aldrich (MAK052, MAK055) (Sigma-Aldrich, St. Louis, MO, USA) and used according to the manufacturer’s instructions. Serum samples were characterized in duplicate for both assays. The absorbance was obtained by a microplate reader at 570 nM for ALT and 450 nM for AST.

### 2.13. Immunoprecipitation and Western Blot

Soluble and insoluble proteins were extracted from cultured cells and mouse livers as previously described [19]. In brief, the samples were sonic homogenized in ice cold, non-denaturing protein extraction buffer. Samples were centrifuged at 10,000× *g* for 30 min at 4 °C. The supernatant, containing soluble protein, and the cell pellet, containing insoluble protein, were retained. The pellet was washed with 1× PBS and centrifuged at 10,000× *g* for 20 min at 4 °C prior to re-suspension in 1% sodium dodecyl sulfate. Protein concentration was determined by bicinchoninic acid protein assay (Thermo Fisher, Waltham, MA, USA).

Soluble protein (500 μg) and insoluble protein (100 μg) were immunoprecipitated (Thermo Fisher, Waltham, MA, USA) with an anti-mouse ubiquitin antibody (Table S1). Antigen precipitated with ubiquitin and its supernatant were loaded onto a 4–20% sodium dodecyl sulfate polyacrylamide gel (SDS-PAGE). The gels were run for 1 h at 100 V. Proteins were then transferred to a 0.2 µM polyvinylidene difluoride (PVDF) membrane for 45 min at 75 V. PVDF membranes were blocked with 5% BSA diluted in Tris-buffered saline with 0.05% tween-20 (TBST) for 1 h. After blocking, membranes were incubated overnight with an anti-human AAT antibody (ubiquitin precipitated protein) or anti-mouse β-actin (supernatant protein as a loading control) (Table S1) at 4 °C, followed by incubation with horseradish peroxidase-conjugated secondary antibodies at room temperature for 1 h (Table S1). Protein bands were visualized by Amersham Imager 680 (GE Healthcare Life Sciences, Chicago, IL, USA) and analyzed by densitometry using Quantity One software (Bio-Rad Laboratories Inc., CA, USA). Other than for immunoprecipitation with ubiquitin antibody, 5–20 μg soluble and insoluble protein were used in direct Western blot with similar protocols. Antibodies against human AAT, mouse albumin, LC3, collagen I, collagen III and β-actin were employed as primary antibodies (Table S1).

### 2.14. Total RNA Extraction and Real-Time RT-PCR

Total RNA was extracted from mouse livers using Trizol reagent per the manufacturer’s protocol (Thermo Fisher, Waltham, MA, USA). After determining RNA concentration via NanoDrop (Thermo Fisher, Waltham, MA, USA), genomic DNA was removed by DNase and cDNA was synthesized using iScript gDNA clear cDNA synthesis kit per the manufacturer’s instructions (BIO-RAD Laboratories Inc., Hercules, CA, USA). The PCR reaction mixture contained: 10 μL Ssofast EvaGreen supermix (BIO-RAD Laboratories Inc., Hercules, CA, USA), 1 μL forward primers (500 nM), 1 μL reverse primers (500 nM), 1 μL cDNA (50 ng RNA), and 7 μL PCR-grade water. The reactions were performed on CFX96 Real-Time PCR Detection System (BIO-RAD Laboratories Inc., Hercules, CA, USA) using the following protocol: 95 °C for 30 s, 40 cycles of 95 °C for 5 s, and 60 °C for 5 s. β-actin was used as an internal control to normalize the amount of input RNA. The primers were synthesized by Invitrogen and the sequences are as follows: β-actin (Gene bank accession number: NM_007393.5) primers 5′-GTGGATCAGCAAGCAGGAGTA-3′ (forward) and 5′-AGGGTGTAAAACGCAGCTC-3′ (reverse) (amplicon size: 96 bp); hAAT (Gene bank accession number: K01396.1) primers 5- GGAGATGCTGCCCAGAAGAC-3′ (forward) and 5′-GCTGGCGGTATAGGCTGAAG-3′ (reverse) (amplicon size: 109 bp) [20]. The relative mRNA expression levels were calculated by the cycle threshold method (delta–delta CT). Standard curves of each pair of primers were established to evaluate the efficiency of the amplification. The amplified sequences were visualized by electrophoresis in 2% agarose gels to verify amplicon size.

### 2.15. Statistical Analyses

Statistical analysis was performed using Prism 7 (GraphPad Software). All results are expressed as mean ± SEM. Body weight and serum data were compared by one-way ANOVA. All other in vitro and in vivo experimental data from treated and control groups were compared using two-sample independent t-tests. For all analyses, *p* values < 0.05 were considered statistically significant.

## 3. Results

### 3.1. Identification of Candidate Compounds by Molecular Docking to the Polymerization Interface of Cleaved AAT

**Figure 1:**
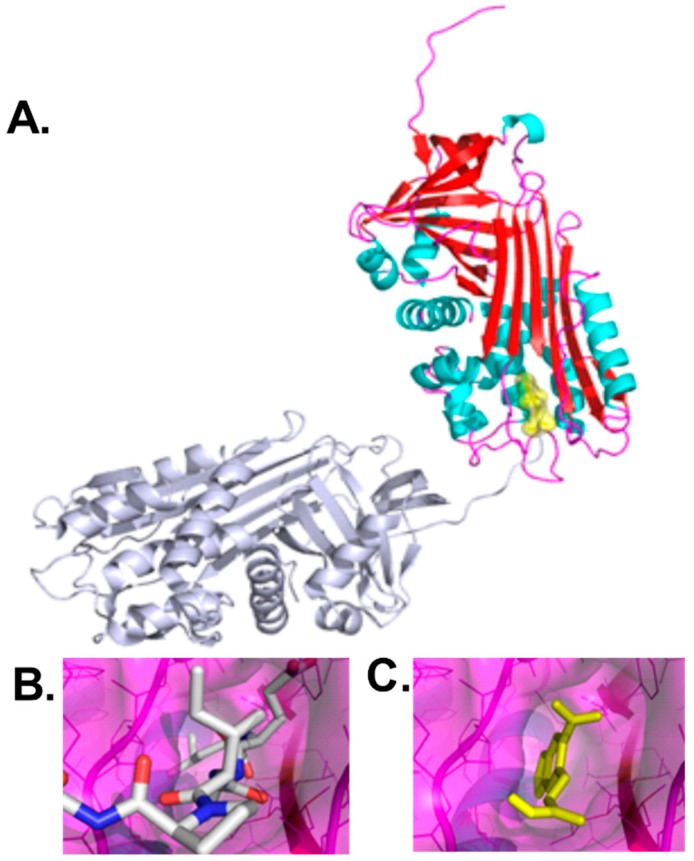
A crystal structure provided the basis for selection of small molecules to inhibit Z-AAT aggregation in hepatocytes. (**A**) The cleaved form of AAT [4] polymerized by intermolecular interactions between a polymerization interface of one AAT molecule: red for β-strands, blue for helices, magenta for loop regions (interacting positions 177–185 and 328–352 in 1QMB), and carboxy terminal tail of an interacting AAT molecule (positions 353–357), shown in gray. A small molecule selected by molecular docking to the polymerization interface, UFC1, is shown in yellow. (**B**) The polymerization interface is shown in which the orientation shown in (**A**) was rotated 90° about a horizontal axis in the plane of the page. The carboxy terminal tail of an interacting AAT molecule is shown as sticks, gray for carbon, blue for nitrogen, red for oxygen. (**C**) Compound UFC1 is shown in yellow as posed by molecular docking using AutoDock Vina.

To identify drug-like small molecules that reduce the intracellular accumulation of polymerized Z-AAT, molecular docking was performed to identify compounds that bind an AAT polymerization interface (Figure 1). The crystal structure of an alpha 1-antitrypsin polymer (PDB 1QMB) revealed an intermolecular linkage by insertion of residues 353–356 (corresponding to P6–P3 of the reactive center loop) of one molecule into the partially occupied β-sheet A of another, resulting in polymer formation [4]. The concave structural pocket accommodating residues 353–356 was selected as the basis for molecular docking (Figure 1). This structural cavity at the polymerization interface consisted of amino acids located in strand 4A, strand 3A, and helix F (positions 177–185 and 328–352 in 1QMB). Each drug-like compound in the National Cancer Institute Developmental Therapeutics Program [16] repository (139,735 in the 2007 Plated Set) was docked in 1000 orientations and scored for polar and non-polar interactions with the polymerization interface cavity. The 20 top scoring compounds were obtained for functional testing.

### 3.2. 4,5-Methylenedioxy-2-nitrocinnamic acid (UFC1) Reduced Accumulation of Intracellular Z-hAAT In Vitro

**Figure 2:**
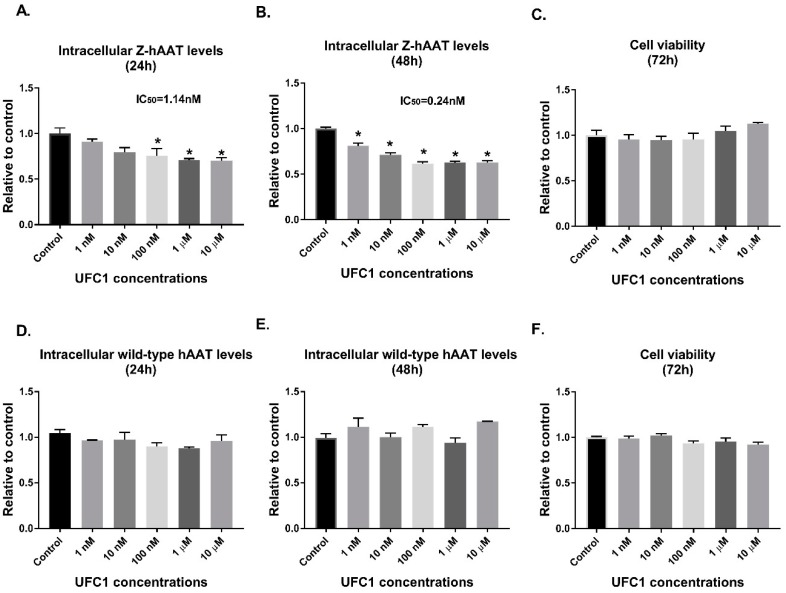
In vitro effects of UFC1 treatment in Z-hAAT hepatocytes: (**A**,**B**) UFC1 reduced intracellular human AAT protein levels in Z-hAAT hepatocytes; (**C**) UFC1 had no effects on cell viability in Z-hAAT hepatocytes; (**D**,**E**) UFC1 had no significant effects on intracellular human AAT protein levels in HepG2 cells; and (**F**) UFC1 had no effects on cell viability in HepG2 cells. Data are presented as mean ± SEM. * *p* < 0.05 relative to control.

Each of the top scoring small molecules predicted to bind the polymerization interface of Z-hAAT were tested for effects on intracellular accumulation and cellular toxicity using the Z-hAAT hepatocyte cell line. 4,5-methylenedioxy-2-nitrocinnamic acid (CID 5702850; termed UFC1) was observed to be effective in reducing the intracellular concentration of Z-hAAT in a potent manner (Figure 2) without affecting cell viability. The extracellular Z-hAAT levels were also decreased (Figure S1).

### 3.3. UFC1 Co-localizes with Z-hAAT in Hepatocytes

**Figure 3:**
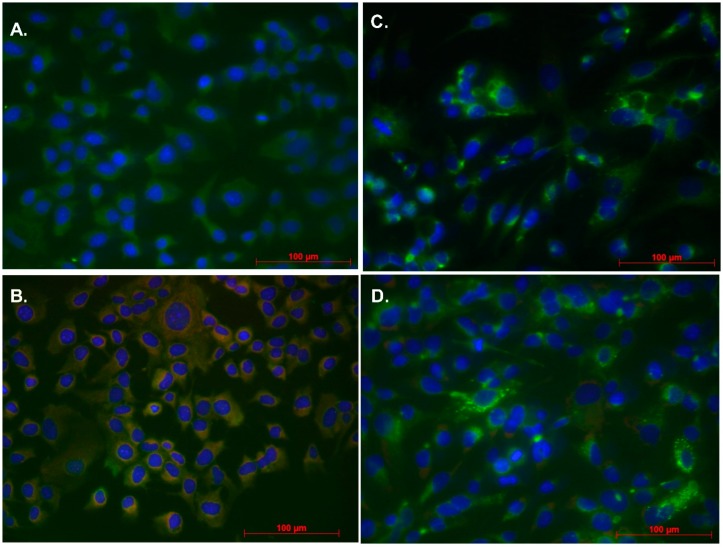
Biotin tagged UFC1 co-localized with Z-hAAT not wild-type hAAT in hepatocytes: (**A**) 10 µM UFC1 cultured with Z-hAAT hepatocytes for 24 h; (**B**) 10 µM biotin tagged UFC1 cultured with Z-hAAT hepatocytes for 24 h; (**C**) 10 µM UFC1 cultured with HepG2 cells for 24 h; and (**D**) 10 µM biotin tagged UFC1 cultured with HepG2 cells for 24 h. hAAT, green color (first antibody, rabbit anti-human AAT; second antibody, anti-rabbit IgG, Alexa Fluor 555). Biotin tagged UFC1, red color (Streptavidin-TMR). hAAT overlapping with biotin tagged UFC1, orange color.

To determine if UFC1 bound and co-localized with hAAT in hepatocytes, UFC1 conjugated with biotin tags were used in double stain immunofluorescence assays. Fluorescence emission of biotin conjugated compounds and hAAT protein in Z-hAAT hepatocytes and HepG2 cells were compared. Unlike HepG2, which showed diffuse expression of UFC1-biotin in cells, Z-hAAT hepatocytes exhibited overlapping detection of Z-hAAT and biotin conjugated UFC1, which predominantly distributed around the cell nucleus (Figure 3 and Figure S2). In contrast, there was no overlapping signal in Z-hAAT hepatocytes and HepG2 cells treated with biotin alone (Figure 3 and Figure S2). Cell viability and UFC1 effects on reducing intracellular Z-hAAT were unaffected with biotin label (Figure S3). These data demonstrate the biotin labeled UFC1 co-localized with Z-hAAT.

### 3.4. UFC1 Decreased Liver Z-hAAT Polymer Aggregation and Reduced Fibrosis In Vivo

**Figure 4:**
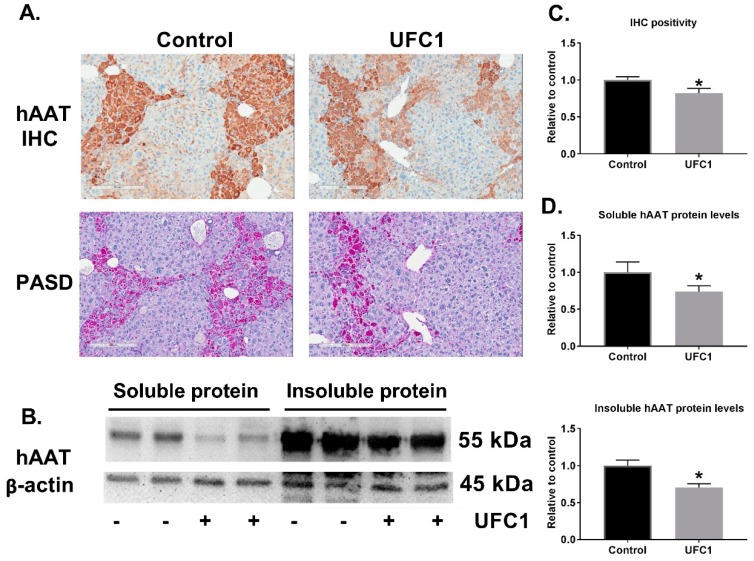
UFC1 treatment reduced Z-hAAT accumulation in PiZ mice livers. Eight-week-old female PiZ mice (n = 7) and age- and gender-matched control PiZ mice (n = 7) were treated for three months with UFC1 (5 mg/kg/day) and PBS, respectively. PASD stain of PiZ mouse liver. (**A**) IHC stain of PiZ mouse liver with an antibody raised against Z-hAAT polymer. Scale bar, 200 µm. (**B**) Soluble and insoluble hAAT protein levels of PiZ mice livers analyzed by Western blot. β-actin was used as a loading control. (**C**) Quantitation of IHC positivity by positive pixel count. (**D**) Quantitation of soluble and insoluble hAAT protein levels from PiZ mice livers. Data are presented as mean ± SEM. * *p* < 0.05 relative to control.

**Figure 5:**
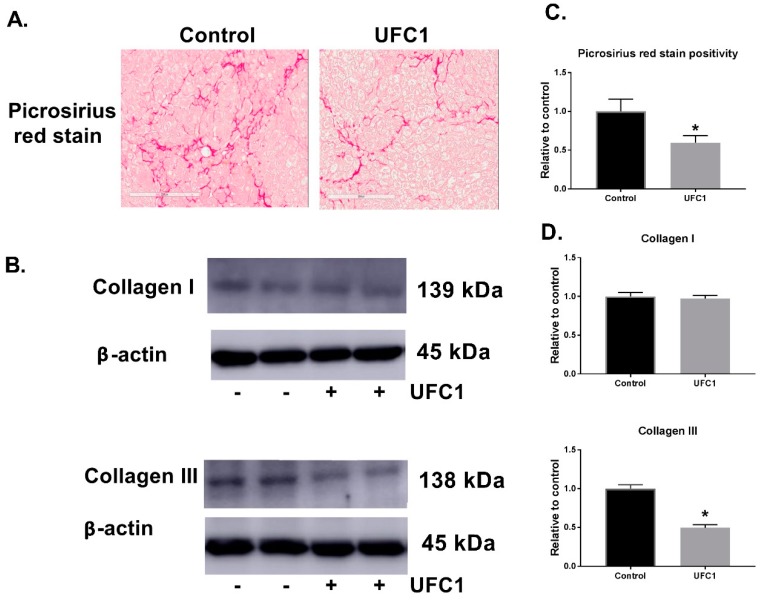
UFC1 treatment alleviated fibrosis in PiZ mice livers. (**A**) Picrosirius red stain of PiZ mice livers. Scale bar, 200 µm. (**B**) Collagen I and collagen III protein levels of PiZ mice livers analyzed by Western blot. β-actin was used as a loading control. (**C**) Quantitation of picrosirius red positivity from PiZ mice livers. (**D**) Quantitation of collagen I and collagen III protein levels from PiZ mice livers. Data are presented as mean ± SEM. * *p* < 0.05 relative to control.

The in vivo effects of UFC1 in PiZ transgenic mice expressing Z-hAAT were investigated. Based on solubility, DMSO concentration, and injection volume, 5 mg/kg was determined to be the highest testable dose. Animals were treated with drug or vehicle control for two weeks, one month, and three months. No significant changes in intracellular Z-hAAT levels were detected by immunohistochemistry or Western blot using antibodies specific for Z-hAAT after two-week treatment (Table 1), and a small reduction of intracellular Z-hAAT was observed after one-month treatment by Western blot, but not by immunohistochemistry (Table 1). Following three months of treatment with UFC1, decreased intracellular Z-hAAT levels were detected by both immunohistochemistry and Western blot (Table 1, Figure 4, and Figure S4). Liver fibrosis, measured by picrosirius red stain and collagen III protein levels were reduced after three months of UFC1 treatment (Figure 5). Mice appeared grossly normal in terms of health during the testing period. No obvious histopathological changes in major organs were observed in both groups. No significant differences in body weight or liver enzyme activity were observed between treated and control groups (Figures S5 and S6).

### 3.5. Specific Effects of UFC1 in Reducing Intracellular Z-hAAT Protein Levels In Vitro and In Vivo

**Figure 6:**
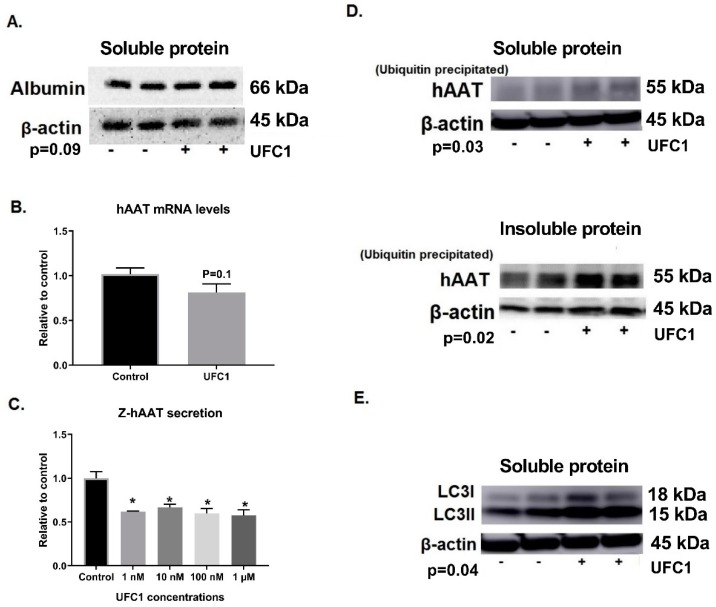
Specificity and mechanism of action of UFC1 in vitro. (**A**) Albumin protein levels were not changed in Z-hAAT hepatocytes after 48 h UFC1 (10 µM) treatment. (**B**) hAAT mRNA levels were not altered in Z-hAAT hepatocytes after 48 h UFC1 (10 µM) treatment. (**C**) Twenty-four-hour UFC1 treatment reduced release of Z-hAAT in hAAT knockout Huh 7.5 hepatoma cells transfected with a tetracycline (Tet) inducible luciferase-tagged Z-hAAT plasmid. (**D**) Forty-eight-hour UFC1 (10 µM) treatment increased soluble and insoluble ubiquitinated hAAT protein levels in Z-hAAT hepatocytes. (**E**) Forty-eight-hour UFC1 (10 µM) treatment increased LC3 II protein levels in Z-hAAT hepatocytes. Data arepresented as mean ± SEM. * *p* < 0.05 relative to control.

**Figure 7:**
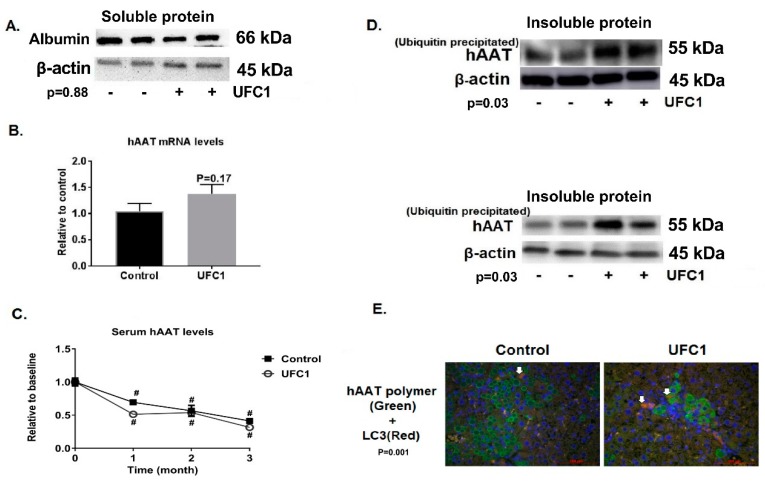
Specificity and mechanism of action of UFC1 in vivo. (**A**) Albumin protein levels were not changed in PiZ mice livers after three-month UFC1 treatment. (**B**) hAAT mRNA levels were not altered in PiZ mice livers after three-month UFC1 treatment. (**C**) Serum hAAT levels in PiZ mice gradually decreased in both UFC1 treated and control groups. No significant change was observed between groups. (**D**) Three-month UFC1 treatment increased soluble and insoluble ubiquitinated hAAT protein in PiZ mice livers. (**E**) Three-month UFC1 treatment increased autophagosomes (LC3 positive staining, white arrows) expression in PiZ mice livers. Scale bar, 100 µm. Data are presented as mean ± SEM. # indicates *p* < 0.05 relative to baseline. Arrow heads indicate LC3 positive staining.

To determine if the protein reducing effects of UFC1 were specific for Z-hAAT, expression of wild-type hAAT in HepG2 and mouse albumin levels in Z-hAAT hepatocytes and in PiZ mice livers were examined. UFC1 did not alter hAAT protein levels in HepG2 cells (Figure 2). Albumin protein levels were unaffected by treatment with UFC1 in vitro (Figure 6A and Figure S7A) and in vivo (Figure 7A and Figure S8A).

### 3.6. UFC1 did not Inhibit Heat-Induced Z-hAAT Polymerization

Unlike compound CG [18], which inhibited heat-induced Z-hAAT polymerization in the in vitro test tube assay, UFC1 did not have this effect at the concentration of 50 and 100 molar fold of Z-AAT (Figure S9).

### 3.7. Z-hAAT Gene Expression was Unaffected by UFC1 Treatment In Vitro and In Vivo

To determine if the compound reduced hAAT protein levels by suppressing hAAT gene expression, Z-hAAT mRNA levels were measured by RT-PCR. No differences were observed between treated and control groups when hAAT mRNA levels were examined in Z-hAAT hepatocytes or PiZ mouse liver (Figure 6B and Figure 7B).

### 3.8. Effects of UFC1 on Z-hAAT Secretion

To determine if UFC1 treatment altered Z-hAAT secretion in vitro, an inducible luciferase-tagged Z-hAAT vector was transiently transfected into hAAT knockout Huh 7.5 hepatoma cells. At concentrations between 1 nM and 1 μM, UFC1 inhibited release of Z-hAAT relative to controls (Figure 6C).

To assay in vivo effects on Z-hAAT secretion, serum Z-hAAT levels of PiZ mice were measured at baseline and following one month, two months, and three months of UFC1 treatment. Serum Z-hAAT levels were found to be reduced at the three treatment time points in both treated and control mice; however, no significant differences were observed between the two groups (Figure 7C).

### 3.9. UFC1 Induced Z-hAAT Ubiquitination In Vitro and In Vivo

Ubiquitinated proteins were immunoprecipitated from mouse Z-hAAT hepatocytes and PiZ mice livers with an antibody raised against mouse ubiquitin. Western blotting was performed using an anti-hAAT antibody to identify immunoprecipitated soluble and insoluble hAAT. Both soluble and insoluble ubiquitinated hAAT protein levels were found to be increased relative to controls in vitro and in vivo (Figure 6D and Figures S7B–D and S8B,C).

### 3.10. UFC1 Enhanced Z-hAAT Degradation through the Autophagic Pathway In Vitro and In Vivo

An anti-mouse LC3I/II antibody was used in Western blotting to examine the degradation of hAAT by the autophagic pathway in vitro. LC3 II intensity was increased after UFC1 treatment in vitro (Figure 6E and Figure S7D). Since lysosomal inhibitors were not administered in PiZ mice, liver LC3 intensity of the bands as detected by Western blot was weak. Alternatively, double stain immunofluorescence was used to visualize hAAT polymer and LC3 positive autophagosomes in PiZ mice livers. The mice were not starved before sacrificing; therefore, very few autophagosomes were visualized [17]. However, in hepatocytes with decreased hAAT globules (polymers), autophagosomes were increased after UFC1 treatment (Figure 7E and Figures S8D and S10).

## 4. Discussion

Since accumulation of mutant AAT at hepatic sites is considered a primary mechanism for proteotoxicity associated with AATD, several strategies have been attempted to inhibit aggregation of mutant AAT molecules [13,14,15,17,18,20]. Chaperone modulating compounds such as PBA were shown to increase Z-hAAT secretion in vitro and in PiZ mice [21,22]. As there is evidence showing that aggregated polymers are degraded by autophagy, interventions to enhance autophagic degradation of aggregation prone proteins are under investigation [17,23]. Gene therapy is a promising strategy to treat AATD [20,24], although approved approaches are currently unavailable [25,26].

Previous attempts have been made to define drug-like small molecules that bind AAT with two important goals: (1) reducing the toxic effects of polymerized AAT in hepatocytes; and (2) enhancing the export of intact active AAT to promote lung protection from neutrophil protease. The topography of intact AAT was evaluated for rational drug design and druggable structural pockets were identified and subsequently used to identify compounds that stabilize AAT [18]. These data demonstrated that AAT binding small molecules can be selected based on solved crystal structures.

Although previous studies were limited by lack of high-resolution structures of the polymerized AAT form most relevant for AATD, Z-AAT, inferences regarding polymerization interfaces were made based on other AAT forms. Two polymerization arrangements have been described: (1) domain swapping by the carboxy-terminal 34 residues in an AAT trimer (3T1P, 3.9 Å) [12]; and (2) the s4A/s5A domain swap observed in the cleaved linear AAT polymer (1QMB, 2.6 Å) [4].

In the current study, we targeted the polymerization interface of the cleaved AAT polymer, which was comprised of elements from strand 4A, strand 3A, and helix F (positions 177–185 and 328–352 in 1QMB) [4]. This structural pocket forms intermolecular interactions with a symmetry related AAT molecule, residues 353–357. Small molecules binding this interface would be expected to inhibit polymerization by steric hindrance of the AAT chain residues 353–357. Molecular docking was utilized to identify and select compounds predicted to bind at the polymerization interface and provide steric hindrance to disfavor polymerization.

We previously developed a cell line comprised of malignant hepatocytes that stably expresses Z-hAAT generated from a PiZ mouse liver for testing candidate compounds for effects on intracellular Z-hAAT aggregation [9]. This cell line resembled disease characteristics demonstrated by PASD resistant staining and positive staining by an antibody raised against Z-hAAT polymer in immunohistochemistry stain. The ratio of intracellular and extracellular hAAT levels in this cell line is consistent with clinical data [9]. In contrast, the human hepatoma cell line HepG2 was used as a negative control since it lacks PASD resistant staining and Z-hAAT polymers, and the majority of the hAAT in HepG2 cells is secreted, which suggests HepG2 cells express wild-type human AAT [9,20,22]. PASD, hAAT, or hAAT polymer were not detected in livers from C57BL/6J mice, confirming the specificity of the assays for Z-hAAT [9]. This PiZ mouse derived cell line allowed candidate compound screening for activity and cytotoxicity in vitro, allowing identification of lead compounds that may translate into safe and effective compounds in vivo.

We tested top scoring small molecules (selected by docking at the AAT polymerization interface) in the Z-hAAT hepatocytes. 4′,′5-(Methylenedioxy)-2-nitrocinnamic acid, termed UFC1, was shown to reduce the intracellular Z-hAAT protein levels, while exhibiting no significant toxicity or proliferation effects. UFC1 did not change albumin protein levels in Z-hAAT hepatocytes and did not alter the intracellular levels of wild-type hAAT in human HepG2 cells. These data suggest that the compound specifically inhibited intracellular accumulation of Z-hAAT in hepatocytes.

To determine if UFC1 bound directly with Z-hAAT in hepatocytes, we labeled UFC1 with a biotin tag and employed the method of double stain immunofluorescence to investigate whether the positive stain of biotin labeled UFC1 overlaps with the stain of hAAT in Z-hAAT hepatocytes and in HepG2 cells. Our results show that biotin labeled UFC1 co-localized with Z-hAAT in Z-hAAT hepatocytes but not wild-type hAAT in HepG2 cells. More importantly, the overlapping signal of biotinylated UFC1 with Z-hAAT was found to be predominantly in close proximity to the cell nucleus, where Z-hAAT aggregates as polymers in the endoplasmic reticulum [7,17].

The in vivo data from PiZ mice were consistent with in vitro data, in which IHC stain and Western blot analysis demonstrated that UFC1 treatment reduced the liver Z-hAAT retention in a time dependent manner. Mice body weight, liver enzymes, and major organs histology were not changed after treatment when compared with controls, which indicates UFC1 was safe to use in mice for a three-month period.

Since fibrosis is a significant problem associated with AATD, we examined the effect of UFC1 on fibrosis in vivo. Picrosirius red stain positivity and collagen III protein levels, which reveal the fibrotic area [27] and fibrosis collagen content [20], respectively, were shown to be reduced significantly in UFC1 treated mice livers.

Although UFC1 was predicted to bind at polymerization interface and inhibit AAT polymerization, there are at least four mechanisms that could contribute to decreased levels of intracellular Z-hAAT protein levels: (1) diminution of Z-hAAT gene expression; (2) increasing protein degradation; (3) increasing Z-hAAT secretion; or (4) inhibiting polymerization and allowing monomer secretion instead of trapping in the cells. Since inhibiting polymerization was our primary target, we incubated Z-hAAT monomers with UFC1 and heated the protein up with lead compounds at different molar folds relative to the protein at 41 °C for seven days; however, we did not see any reduction in heat-induced polymer formation with this assay.

Heat-induced Z-hAAT polymerization assay (an in vitro test tube assay) has been used to test compounds that may have the potential to inhibit Z-hAAT polymerization in the real disease [12,18]. However, compounds that inhibited heat-induced Z-hAAT polymerization reduced intracellular Z-hAAT levels but did not increase extracellular Z-hAAT levels in a cell model of AATD [18], which may indicate that, instead of being secreted, Z-hAAT monomers/dimers/trimers are more prone to being degraded by the cells (Figure S11). In our study, we did not observe the effect of UFC1 on inhibiting heat-induced polymerization; however, both the intracellular and extracellular Z-hAAT levels are decreased in our cell model of AATD, which suggests UFC1 may inhibit an alternative process of Z-hAAT polymerization. It is also possible that UFC1 increased Z-hAAT degradation with mechanisms other than interrupting polymerization.

We next investigated other possible mechanisms and found that UFC1 had no effect on hAAT gene expression but increased both ubiquitin and autophagy associated Z-hAAT degradation in vitro and in vivo. The secretion of Z-hAAT, as measured in the luciferase-tagged Z-hAAT plasmid expression system in hAAT knock-out Huh 7.5 hepatoma cells, was also reduced, as was extracellular Z-hAAT protein levels in Z-hAAT hepatocytes. This may be a consequence of protein degradation as the secretion of Z-hAAT was decreased without dose-dependency, which may indicate saturated protein degradation ability of the cells (Figure S11). There are two types of AAT secretion: acute-phase secretion in response to inflammation and steady-state secretion [28]. The secretion of Z-hAAT in vitro is expected to be steady-state secretion. If the secretion of serum Z-hAAT in the in vivo study was acute-phase secretion, this may explain why serum Z-hAAT levels in PiZ mice after UFC1 treatment were not changed relative to controls. Therefore, we measured serum inflammatory cytokine levels (IL-6) [29]. The results show that serum IL-6 levels were not detectable with a commercial ELISA kit in both UFC1 treated and control groups (Figure S12), suggesting the serum hAAT measured in both groups was steady-state secretion. The underlying mechanism of differential effects of UFC1 on Z-hAAT secretion in vitro and in vivo remains to be elucidated. We hypothesize that the body may regulate steady-state AAT secretion in response to reduction in intracellular and/or extracellular AAT levels, while the in vitro cultured cells do not have this ability to regulate Z-hAAT secretion (Figure S11). Moreover, PiZ mice express endogenous mouse AAT, which may reflect adaptation of the mice who do not need Z-hAAT and so directly degrade it.

In brief, there are two possible mechanisms of action of UFC1 on reducing intracellular Z-hAAT levels: (1) UFC1 inhibited Z-hAAT polymerization, which is different from the process of heat-induced polymerization. Instead of being secreted, Z-hAAT monomers are degraded by the cells. (2) UFC1 increased the degradation of intracellular Z-hAAT through an unknown mechanism.

In summary, our results suggest that UFC1 (4′,′5-(Methylenedioxy)-2-nitrocinnamic acid) may interact directly with Z-hAAT and render the protein susceptible to degradation mechanisms within cells in a manner beneficial for AATD associated liver disease. AAT substitution will remain necessary to assure antielastase activity.

## Figures and Tables

**Table 1:** Relative hAAT protein expression in PiZ mice liver after UFC1 treatment.

Liver hAAT Protein Expression	Treatment Period								
	Two Weeks			One Month			Three Months		
	Control (n = 6)	UFC1 (n = 6)	*p* Value	Control (n = 5)	UFC1 (n = 6)	*p* Value	Control (n = 7)	UFC1 (n = 7)	*p* Value
**hAAT polymer (IHC)**	1 ± 0.1	1.1 ± 0.1	0.6	1 ± 0.1	0.9 ± 0.1	0.9	1 ± 0.04	0.8 ± 0.1	0.04
**Soluble hAAT protein (WB)**	1 ± 0.1	0.9 ± 0.1	0.5	1 ± 0.01	0.9 ± 0.01	0.02	1 ± 0.1	0.6 ± 0.1	0.03
**Insoluble hAAT protein (WB)**	1.2 ± 0.2	1 ± 0.2	0.7	1 ± 0.01	0.9 ± 0.01	0.02	1 ± 0.1	0.7 ± 0.1	0.007

Data are presented as mean ± SEM. hAAT, human alpha1-antitrypsin; IHC, immunohistochemistry; WB, Western blot.

## Supplementary Materials

The following are available online at https://www.mdpi.com/2073-4409/8/12/1586/s1, Figure S1: Extracellular Z-hAAT levels in Z-hAAT hepatocytes after 24 h treatment with UFC1, Figure S2: Control stains of Figure 7, Figure S3: Biotin tagged UFC1 did not change cell viability and UFC1 effects on reducing intracellular AAT levels in Z-hAAT hepatocytes, Figure S4: Full blots of Figure 3B, Figure S5: Body weight of PiZ mice during UFC1 treatment, Figure S6: Serum ALT and AST activity in PiZ mice during UFC1 treatment, Figure S7: Quantification data of Figure 6, Figure S8: Quantification data of Figure 7, Figure S9: Heat-induced polymerization of Z-hAAT assessed by 7.5% (w/v) nondenaturing PAGE, Figure S10: Figure S6: Serum ALT and AST activity in PiZ mice during UFC1 treatment, Figure S11: Graphical hypothesis of mechanism UFC1 effects on intracellular and extracellular Z-hAAT levels, Figure S12: Serum concentration of IL-6, Table S1: Antibodies used in methods.

## Appendix A

### Synthesis of Tag-Biotin with Compound UFC1

Synthesis of Tag-biotin: 3-(3,4-methylenedioxy-6-nitrophenyl)propenoic acid (c1, 0.47 g, 2 mmol) was dissolved in DMF (10 mL) containing trimethylamine (TEA, 0.30 g, 3 mmol), HATU (1.14 g, 3 mmol), and N-BOC-1,2-Diaminoethane (0.40 g, 2.5 mmol). The mixture was stirred at room temperature overnight and poured into water. The product was extracted with ethyl acetate, washed with saturated saline solution, dried over anhydrous Na_2_SO_4_, concentrated, and purified using silica gel column chromatography, yielding a yellow solid (c2, 0.69 g, yield 92%). c2 (0.38 g, 1 mmol) was dissolved in 10 mL trifluoroacetic acid/methylene chloride (TFA/DCM, v/v, 1/1) and stirred for 2 h. The solvent was completely removed by vacuum and the de-protected product was re-dissolved in 5 mL DMF. Then, biotin (0.29 g, 1.2 mmol) in 10 mL DMF containing HATU (0.57 mg, 1.5 mmol) and TEA (0.20 g, 2 mmol) was added. The mixture was stirred for 24 h at room temperature. The DMF solvent was removed by vacuum and the product was purified using silica gel column chromatography, yielding a yellow solid (Tag-biotin, 0.31 g, yield 61%). ^1^H NMR (300 MHz, DMSO-*d*_6_) δ 8.21 (t, *J* = 5.5 Hz, 1H), 7.86 (t, *J* = 5.4 Hz, 1H), 7.71 (t, *J* = 5.6 Hz, 1H), 7.63 (d, *J* = 15.4 Hz, 1H), 7.65 (s, 1H), 7.23 (s, 1H), 6.50 (d, *J* = 15.5 Hz, 1H), 6.41 (s, 1H), 6.34 (s, 1H), 6.25 (s, 2H), 4.37-4.22 (m, 1H), 4.10 (t, *J* = 5.7 Hz, 1H), 3.17–3.07 (m, 2H), 2.97 (q, *J* = 6.6 Hz, 2H), 2.02 (td, *J* = 7.4, 2.6 Hz, 4H), 1.57–1.40 (m, 6H). HRMS (*m/z*): [M + H]^+^ calculated for C_22_H_28_N_5_O_7_S, 506.1665; found, 506.1727.
