# Supplementary material for: A Novel Small Molecule Inhibits Intrahepatocellular Accumulation of Z-Variant Alpha 1-Antitrypsin In Vitro and In Vivo
Source: Cells. 2019 Dec 6;8(12):1586. doi: 10.3390/cells8121586 (PMC6953066; doi:10.3390/cells8121586)
Supplement: Supplementary file 1 [file cells-08-01586-s001.pdf]

Supplemental materials and methods

Hematoxylin and eosin stain

The liver, heart, lung, spleen, kidney and pancreas tissues were fixed in 10% formalin and embedded in paraffin. Tissue sections (4 µm) were de-paraffinized and rehydrated with water. Hematoxylin was applied to the slides. After washing with water, eosin was added, followed by dehydration with ethanol and xylene. The coverslips were then cleared and mounted on slides. The slides were evaluated by a pathologist blindly.

Mouse IL-6 ELISA assay

Mouse IL-6 platinum ELISA kit (Affymetrix eBioscience, BMS603/2TWO) was used to determine serum concentration of IL-6 in control and UFC1 treated mice. The assay was carried out according to the manufacturer's instructions. In brief, the plate was washed with wash buffer, then standards, samples and biotin-conjugate were added to the wells in duplicates and incubated at room temperature for 2 h. After washing, streptavidin-HRP was added and the plate was incubated at room temperature for 1 h. TMB substrate solution was added after final wash. About 30 minutes later, stop solution was pipetting in to each well. The absorbance was read at a wavelength of 490 nm on a spectro-photometer (Molecular Devices, LLC, CA, USA).

Supplemental data

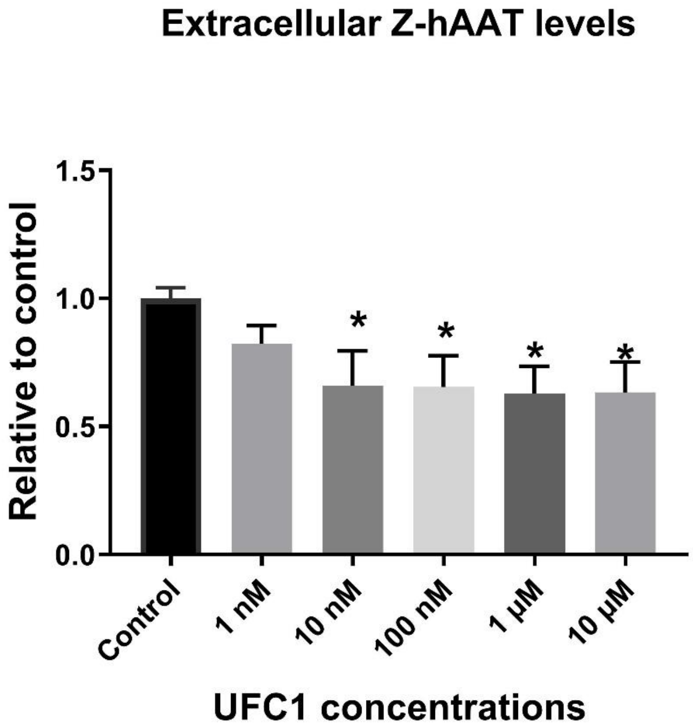

Supplemental Figure S1. Extracellular Z-hAAT levels in Z-hAAT hepatocytes after 24 h treatment with UFC1. Data are represented as mean ± SEM. * $p < 0.05$ relative to control.

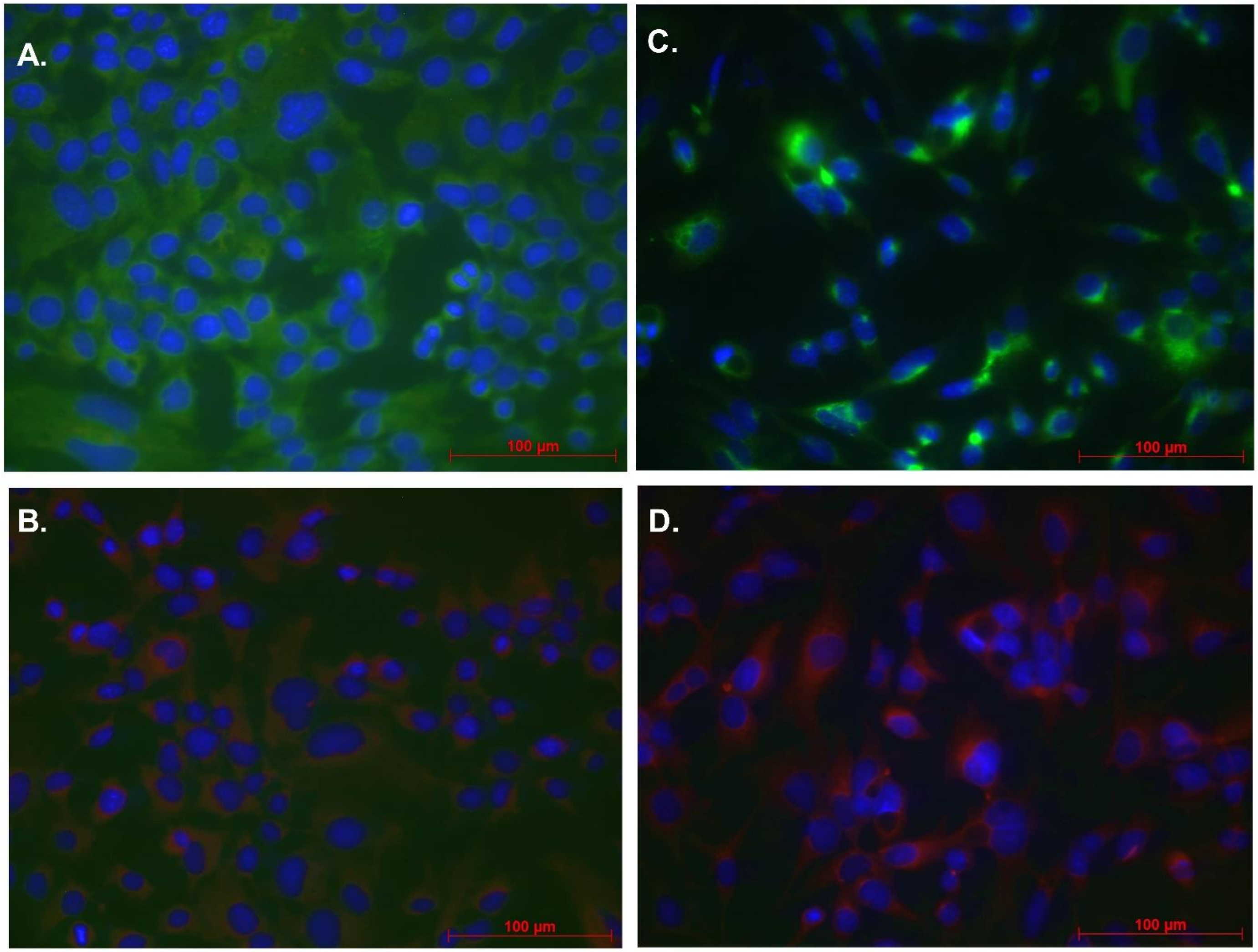

Supplemental Figure S2. Control stains of figure 7. (**A**) 10 µM biotin cultured with Z-hAAT hepatocytes for 24 h. (**B**) Z-hAAT hepatocytes cultured for 24 h. (**C**) 10 µM biotin cultured with HepG2 cells for 24 h. (**D**) HepG2 cells cultured for 24 h. (**A**,**C**) 1st antibody, rabbit anti-human AAT, 2nd antibody, anti-rabbit IgG, Alexa Fluor 555 (green color), followed by streptavidin-TMR. (**B**,**D**) 1st antibody, rabbit anti-human AAT, 2nd antibody, biotin conjugated anti-rabbit IgG, followed by streptavidin-TMR (red color).

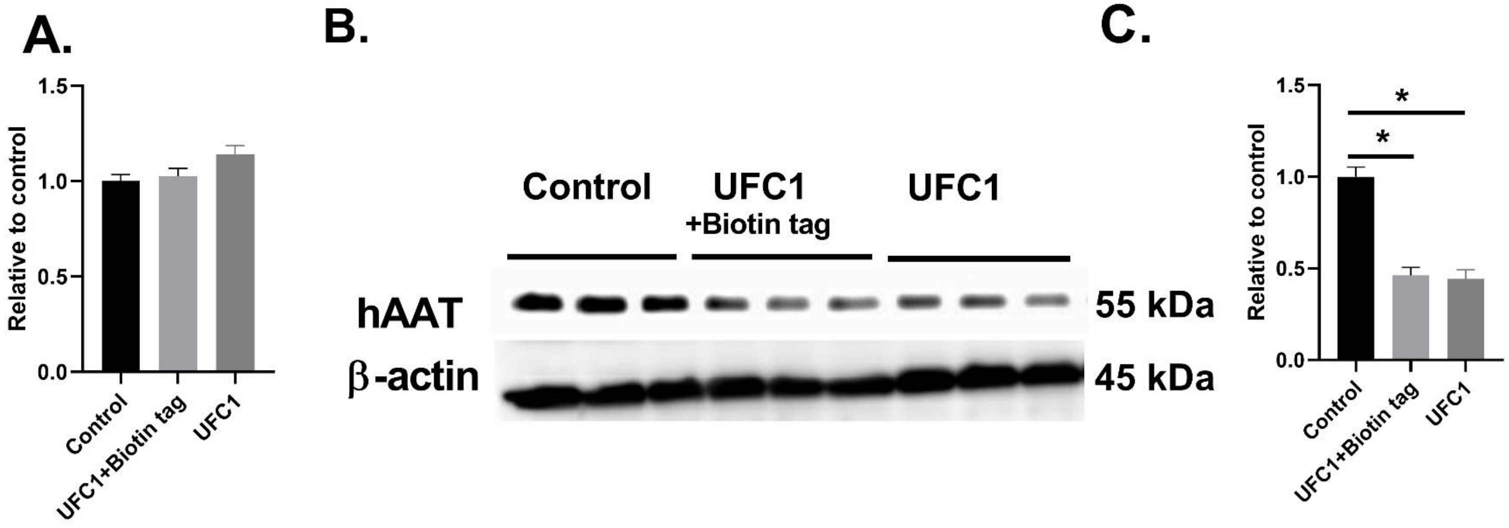

Supplemental Figure S3. Biotin tagged UFC1 did not change cell viability and UFC1 effects on reducing intracellular AAT levels in Z-hAAT hepatocytes. (**A**) Cell viability after 48 h treatment. (**B**) hAAT protein levels after 48 h treatment. (**C**) Quantification B. * $p < 0.05$ relative to control.

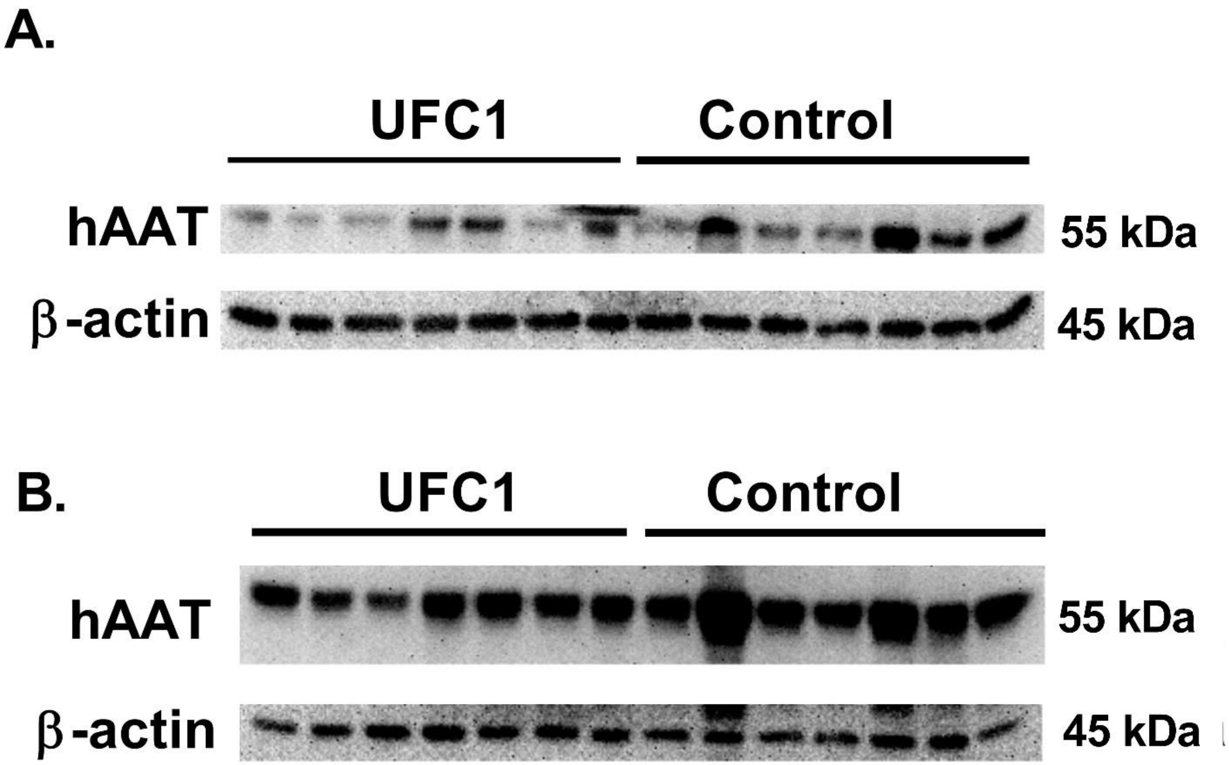

Supplemental Figure S4. Full blots of figure 3B. (**A**) Soluble human AAT protein levels in UFC1 treated and control PiZ mice livers. (**B**) Insoluble human AAT protein levels in UFC1 treated and control PiZ mice livers. AAT, alpha-1 antitrypsin.

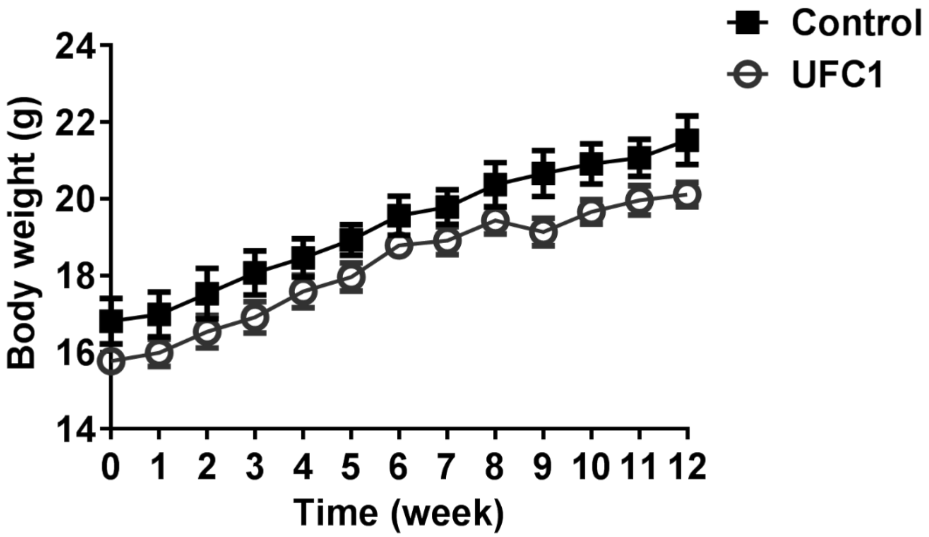

Supplemental Figure S5. Body weight of PiZ mice during UFC1 treatment. Body weight of PiZ mice increased gradually over the 3-month treatment time period, with no significant changes observed between UFC1 treated and control groups.

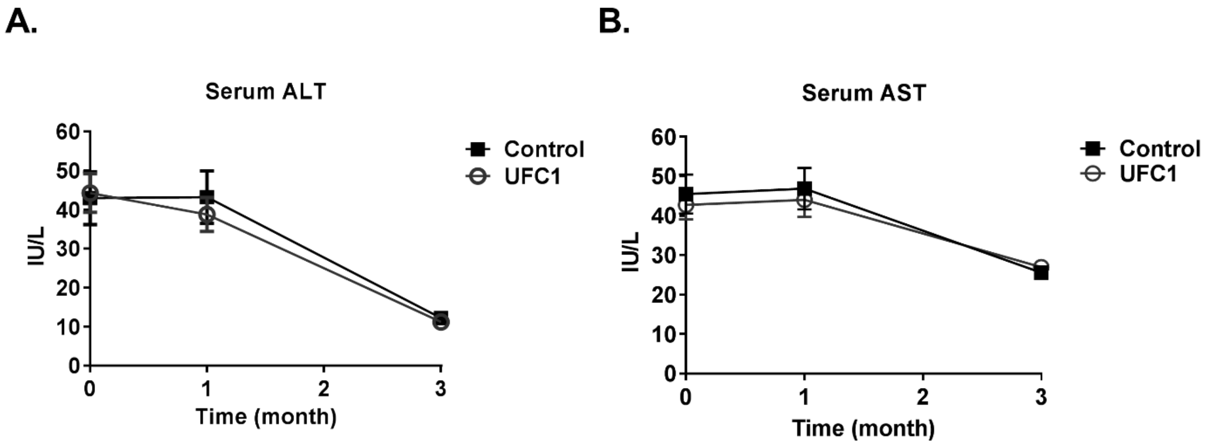

Supplemental Figure S6. Serum ALT and AST activity in PiZ mice during UFC1 treatment. Serum ALT (**A**) and AST (**B**) levels were found to be reduced over the 3-month period in both control and UFC1 treated groups. No significant difference were found between the two groups at each time point.

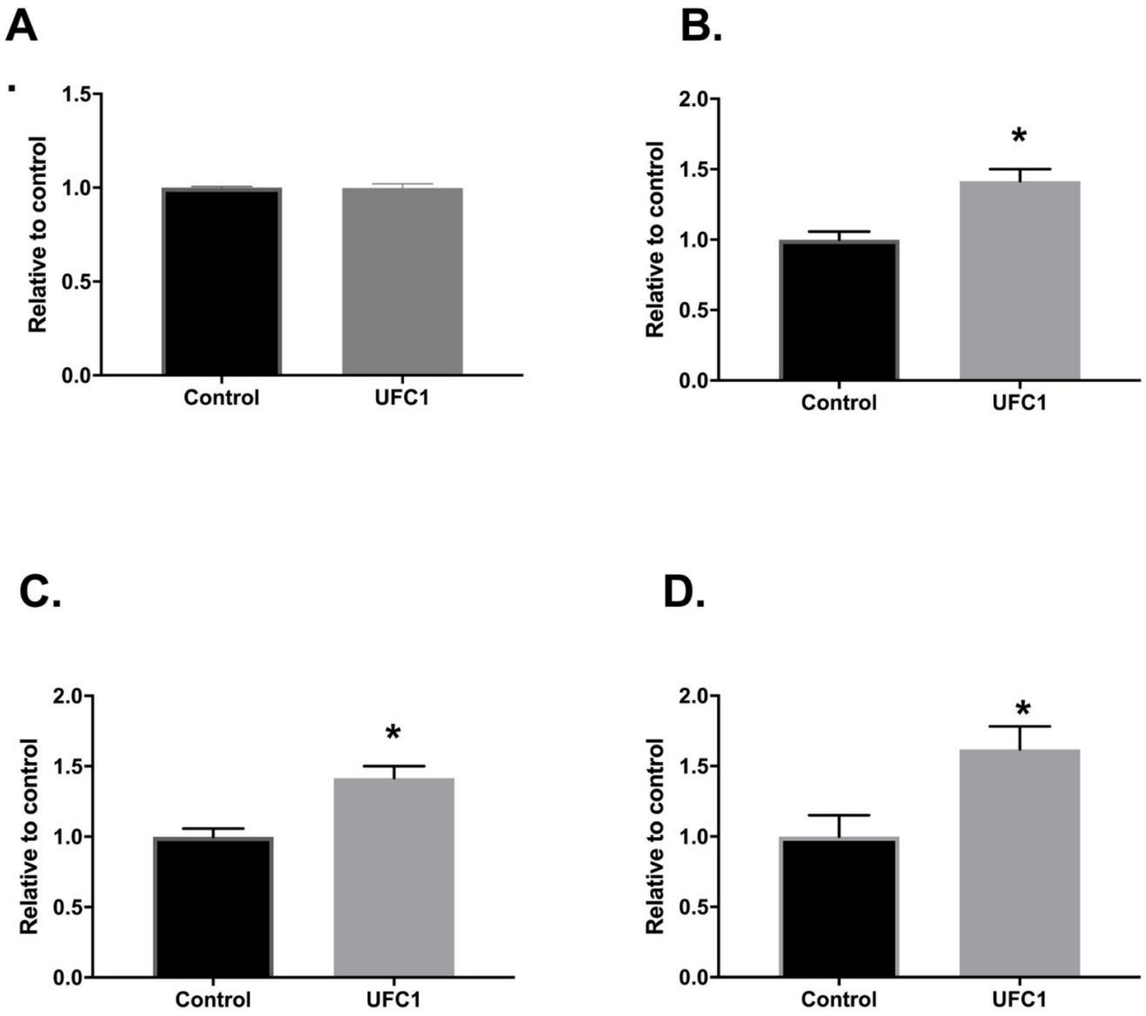

Supplemental Figure S7. Quantification data of figure 5. (**A**) Quantification of figure 5A. Albumin protein levels in UFC1 treated and control Z-hAAT hepatocytes. (**B**) Quantification of figure 5D. Ubiquitin precipitated soluble hAAT protein levels in UFC1 treated and control Z-hAAT hepatocytes. (**C**) Quantification of figure 5D. Ubiquitin precipitated insoluble AAT protein levels in UFC1 treated and control Z-hAAT hepatocytes. (**D**) Quantification of figure 5E. LC3 II protein levels in UFC1 treated and control Z-hAAT hepatocytes. . * $p < 0.05$ relative to control.

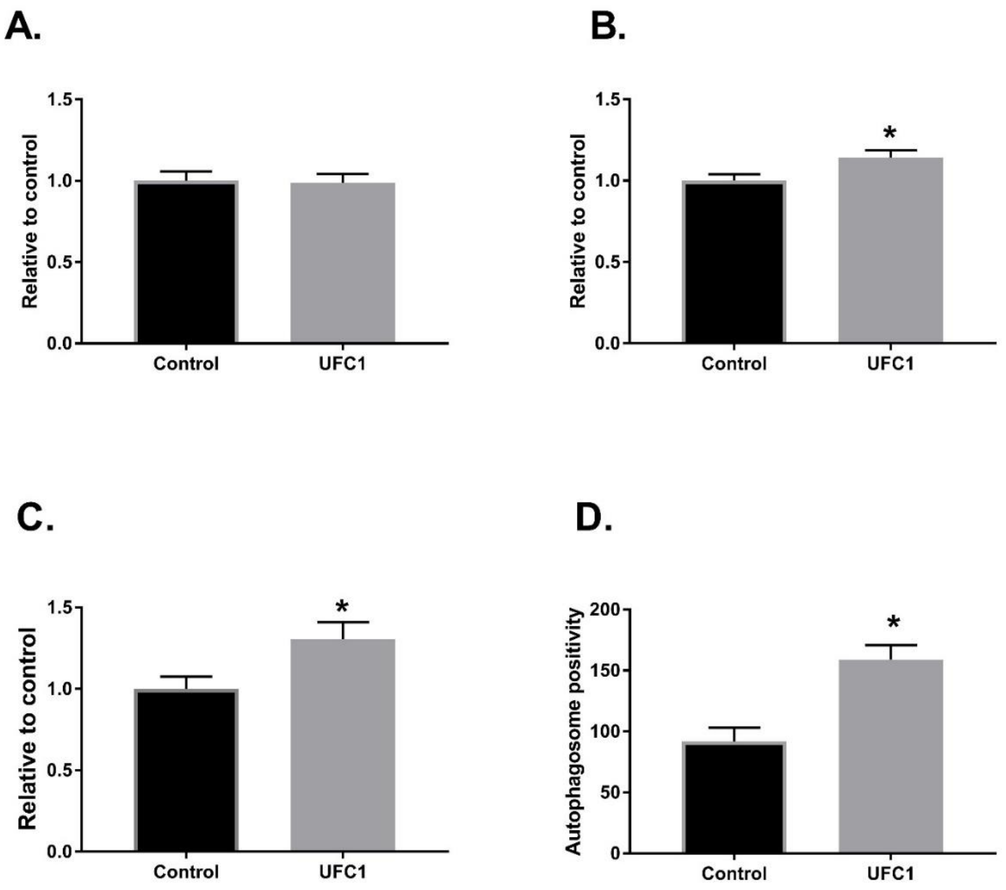

Supplemental Figure S8. Quantification data of figure 6. (**A**) Quantification of figure 6A. Albumin protein levels in UFC1 treated and control PiZ mice livers. (**B**) Quantification of figure 6D. Ubiquitin precipitated soluble AAT protein levels in UFC1 treated and control PiZ mice livers. (**C**) Quantification of figure 6D. Ubiquitin precipitated insoluble AAT protein levels in UFC1 treated and control PiZ mice livers. (**D**) Quantification of figure 5E. LC3 II protein levels in UFC1 treated and control PiZ mice livers. . * $p < 0.05$ relative to control.

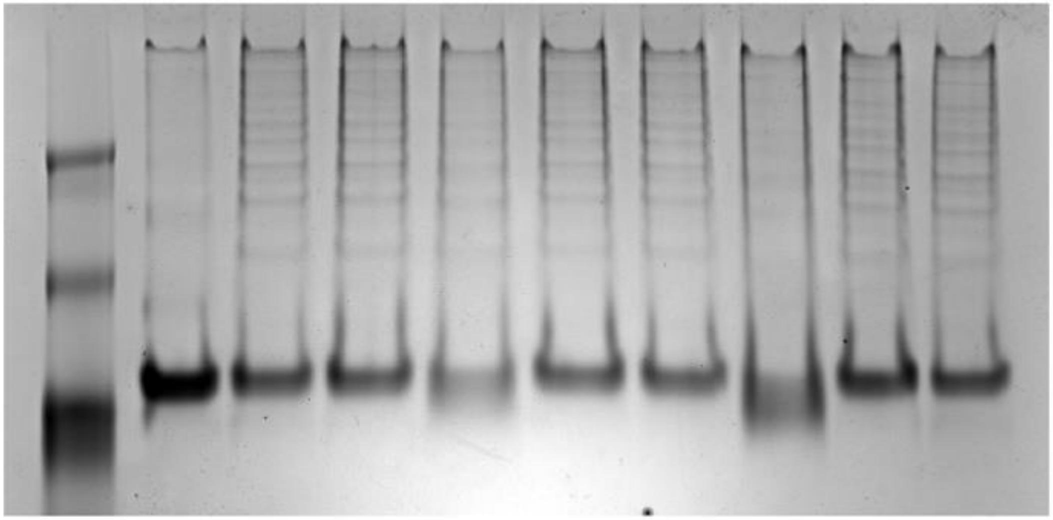

Supplemental Figure S9. Heat-induced polymerization of Z-hAAT assessed by 7.5% (w/v) nondenaturing PAGE. Z-hAAT monomers (0.1 mg/mL) were heated at 41 °C for 7 days with or without compounds of interests. Each lane contains 2 μg of protein. Lane 1, protein marker; Lane 2, wild-type AAT monomer obtained from healthy subjects; Lane 3, Z-AAT; Lane 4, Z-AAT with 4.75% (v/v) DMSO; Lane 5, Z-AAT with 50–fold molar excess of CG; Lane 6, Z-AAT with 50–fold molar excess of UFC1; Lane 7, Z-hAAT with 50–fold molar excess of an unpublished compound; Lane 8, Z-AAT with 100–fold molar excess of CG; Lane 9, Z-hAAT with 100–fold molar excess of UFC1; Lane 10, Z-hAAT with 100–fold molar excess of an unpublished compound.

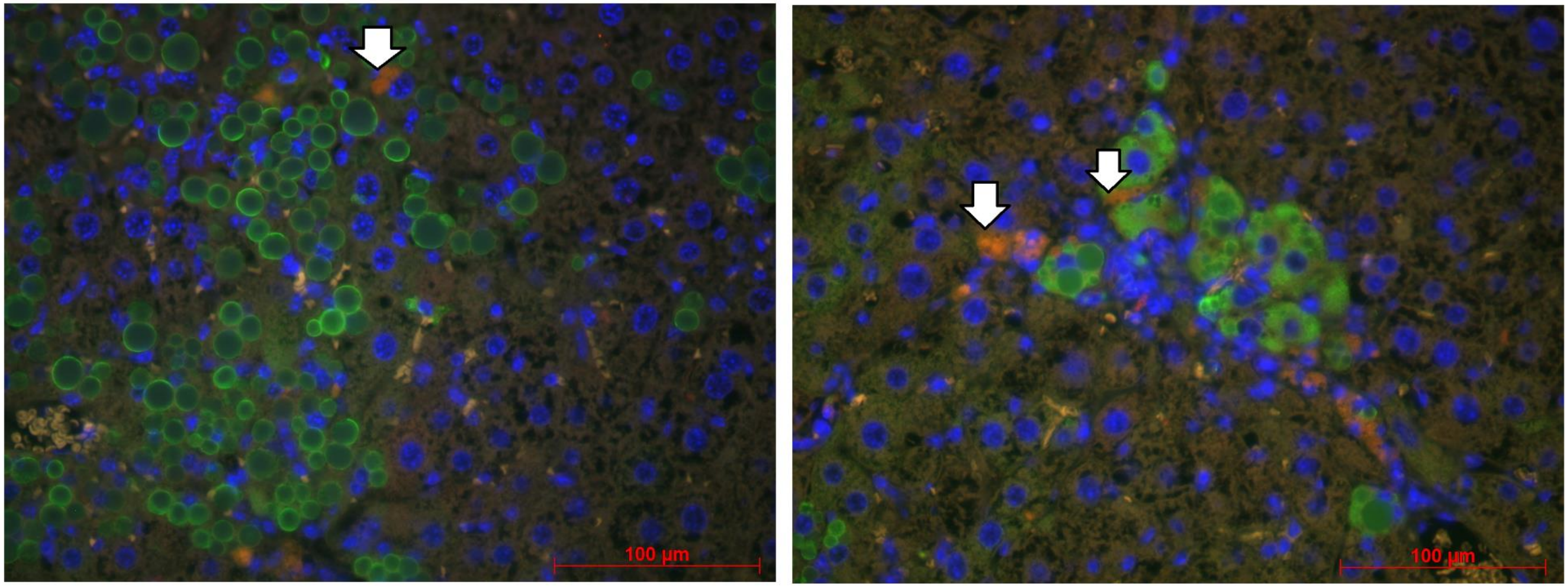

Supplemental Figure S10. Figure 6E. Left panel control group. Right panel, UFC1 group. White arrows, LC3 positive staining.

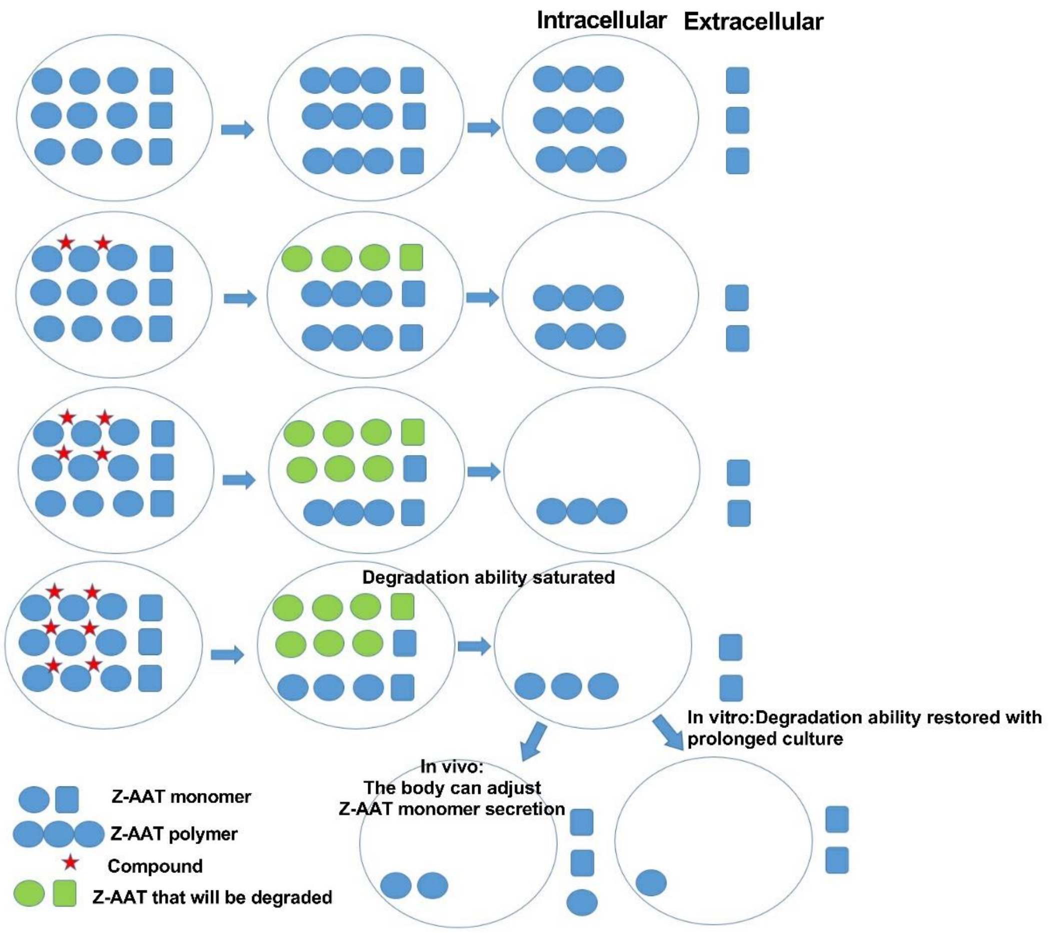

Supplemental Figure S11. Graphical hypothesis of mechanism UFC1 effects on intracellular and extracellular Z-hAAT levels.

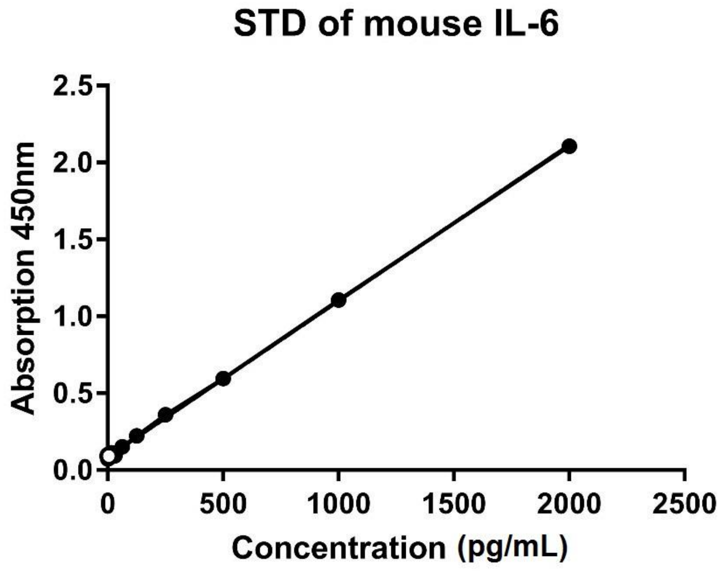

Supplemental Figure S12. Serum concentration of IL-6. The concentration of serum IL-6 from control and UFC1 treated mice dropped at or out of the lower limit of the STD. Black dots represent standards, white dot represents a serum sample that is close to the lower limit of the STD. STD, standard curve.

Supplemental Table S1 Antibodies used in methods.

			Application					Company	Cat #
	Antigen	Host	ELISA	IP	IF	Western blot	IHC		
Primary antibodies	hAAT	Goat	1:500					MP pharmaceuticals	55111
	hAAT	Rabbit	1:1000			1:5000		Sigma-Aldrich	A0409
	hAAT	Rabbit			1:200			Fitzgerald	20R-AR009
	hAAT polymer	Mouse			1:10		1:1000	Hycult biotech	HM2289
	Ubiquitin	Mouse		2 µg				Santa Cruz	Sc-8017
	LC3	Rabbit			1:100	1:1000		Novus Biologicals	NB1002220
	Albumin	Mouse				1:100		Santa Cruz	sc-374670 HRP
	β-actin	Mouse				1:10000		R&D	MAB8929-SP
Secondary antibodies	Anti-rabbit IgG, HRP	Goat	1:2500			1:2000		Sigma-Aldrich	A0545
	Anti-mouse IgG, HRP	Goat				1:1000		R&D	HAF018
	Mach 2 Rabbit HRP polymer	Goat						Biocare Medical	RHRP520
	Mouse on mouse Elite Peroxidase Kit							Vector labs	Pk-2200
	Anti-rabbit IgG, Alexa Fluor 555	Goat			1:500			Lifetechnologies	A21429
	Anti-mouse IgG, Alexa Fluor 488	Goat			1:500			Invitrogen	A11029
	Anti-rabbit IgG, biotin conjugate	Goat			1:1000			Sigma	B7389
	Anti-rabbit IgG, Alexa Fluor 488	Goat			1:500			Invitrogen	A11034
	Streptavidin, tetramethylrhodamine conjugate				1:500			Thermofisher	S870

ELISA, enzyme-linked immunosorbent assay, IP, immunoprecipitation, IF, immunofluorescence, IHC, immunohistochemistry, hAAT, human alpha1-antitrypsin, HRP, horseradish peroxidase.
